# Abrogating ALIX Interactions Results in Stuttering of the ESCRT Machinery

**DOI:** 10.3390/v12091032

**Published:** 2020-09-16

**Authors:** Shilpa Gupta, Mourad Bendjennat, Saveez Saffarian

**Affiliations:** 1 Center for Cell and Genome Sciences, University of Utah, Salt Lake City, UT 84112, USA; u0878193@utah.edu (S.G.); mbendjennat@med.miami.edu (M.B.); 2 Department of Biology, University of Utah, Salt Lake City, UT 84112, USA; 3 Radiation Oncology Department, University of Miami, Miami, FL 33136, USA; 4 Department of Physics and Astronomy, University of Utah, Salt Lake City, UT 84112, USA

**Keywords:** ESCRT, stuttering, HIV, ALIX, CHMP4B, VPS4

## Abstract

Endosomal sorting complexes required for transport (ESCRT) proteins assemble on budding cellular membranes and catalyze their fission. Using live imaging of HIV virions budding from cells, we followed recruitment of ESCRT proteins ALIX, CHMP4B and VPS4. We report that the ESCRT proteins transiently co-localize with virions after completion of virion assembly for durations of 45 ± 30 s. We show that mutagenizing the YP domain of Gag which is the primary ALIX binding site or depleting ALIX from cells results in multiple recruitments of the full ESCRT machinery on the same virion (referred to as stuttering where the number of recruitments to the same virion >3). The stuttering recruitments are approximately 4 ± 3 min apart and have the same stoichiometry of ESCRTs and same residence time (45 ± 30 s) as the single recruitments in wild type interactions. Our observations suggest a role for ALIX during fission and question the linear model of ESCRT recruitment, suggesting instead a more complex co-assembly model.

## 1. Introduction

Endosomal sorting complexes required for transport are cellular proteins that catalyze the fission of membranes and play an important role in biology of diseases, including cancer and infectious virus release [1]. ESCRTs were discovered in *Saccharomyces cerevisiae* when their genetic deletion caused abnormal sorting of cargo in multivesicular bodies [2]. The functioning units of ESCRTs in multivesicular trafficking were further identified in yeast as the VPS4 protein which is a AAA ATPase [3] along with multi-protein complexes: ESCRT-I [4] and ESCRT-II [5], yeast Bro1 [6,7] and ESCRT-III proteins [5]. Based on identified biochemical interactions, the overall mechanism of ESCRT recruitment was proposed to start with cargo proteins binding to ESCRT-I and ESCRT-II proteins. ESCRT-I and ESCRT-II complexes in turn recruit ESCRT-III proteins. ESCRT-III proteins in turn catalyze the fission of membrane in conjunction with VPS4 recruitment [8,9,10].

The discovery of the PTAP sequence on the Gag p6 domain of HIV and its importance in infectious virion release [11,12] initiated a search for its cellular partners. Binding of the PTAP sequence to ESCRT-1 proteins demonstrated the role of ESCRTs in HIV release [13,14,15]. Further, divergence between early ESCRTs in multivesicular bodies and HIV budding was observed: primarily, in the HIV budding pathway the link to ESCRT-II has been controversial; secondly, there is no direct mammalian homologue within the ESCRT pathway to Bro1 protein identified in *Saccharomyces cerevisiae* [16,17,18]. The closest analogue to Bro1 was identified as ALIX which has a Bro domain with similar ESCRT-III interactions as identified in *Saccharomyces cerevisiae*. However. mammalian ALIX, in addition to its Bro domain, has a V domain which binds directly to the YP motif, the second HIV late domain located on the HIV Gag-p6 [19,20,21]. While depletion of the ESCRT-I complex completely abrogates infectivity of released HIV virions, abrogating ALIX interactions were shown to only partially affect the infectivity [22]. In the mammalian system similar to the identified yeast interactions, it was shown that over-expression of ESCRT-III proteins resulted in deformations on the plasma membranes of cells [23], and helical structures of ESCRT-III proteins were depolymerized by VPS4 [24]. The overall mechanism of ESCRT recruitment in HIV budding, however, was proposed with similar step-wise recruitment of ESCRT factors—specifically, Gag late domains recruiting ESCRT-I and ALIX who in turn recruit ESCRT-III proteins which polymerize on the neck of the budding virions and catalyze the membrane fission reaction in conjunction with VPS4 [25,26,27,28].

ALIX and ESCRT-I were shown to bind Cepp55 at the cleavage furrow during cytokinesis; it was further shown that ESCRT-III proteins and VPS4 are also recruited and play an essential role in cytokinesis [29,30,31]. The functional units of ESCRTs in cytokinesis and HIV budding are similar in that they both require ESCRT-I and both rely on ESCRT-III polymers and VPS4. The difference between the two pathways is firstly their scale and secondly the essential role ALIX plays in cytokinesis compared to its peripheral role in HIV budding [32].

In the past decade the number of identified intracellular processes dependent on ESCRTs has increased dramatically and now include exosome release [33,34], down regulation of G-protein coupled receptors [35], plasma membrane repair [36] and nuclear envelope sealing [37]. Understandably, major efforts in structural biology have been made to understand the molecular mechanism of ESCRT function. At present, at least some structural information about almost all proteins within the pathway is available [38]. Reconstructing the ESCRT functions in vitro has also led to identification of ESCRT function in reverse topology membrane fission which has been implicated in endosomal pathway in cells [39].

While ESCRTs are implicated in various cellular processes, the fundamental mechanism of ESCRT function is still assumed to be similar to what was proposed in multivesicular body biogenesis, namely, that early ESCRT-I and possibly ESCRT-II proteins bind the cargo and recruit the ESCRT-III and VPS4 proteins to perform the fission reaction. The details of the molecular mechanism of ESCRT function, including how recruitment is orchestrated, how the ESCRT-III proteins work in conjunction with VPS4 to catalyze the fission and how ATP hydrolysis couples to the membrane fission reaction, however, remain obscure.

The results from live imaging observations of ESCRTs have added to the mystery of ESCRT function. During imaging of multivesicular body biogenesis in yeast cells, ESCRT-III and VPS4 proteins were observed polymerizing and depolymerizing on the membrane before catalyzing the fission reaction, which was surprisingly found to be ATP-independent [40]. During imaging of HIV virus-like particle (VLP) budding from mammalian cells in culture, membrane fission was detected to occur up to a minute after all the ESCRTs had been released back into the cytosol [41].

Aside from live imaging, when kinetic release experiments were performed on release of infectious HIV particles, it was found that abrogating ESCRT interactions did not result in a full blockage of the release of virions as previously assumed. Instead it was shown that virions which had abrogated interactions with early ESCRTs eventually managed to release with a considerable delay. It was further shown that this delay led to an untimely activation of the HIV protease and release of non-infectious virions [42].

Using live imaging we have visualized the recruitment of ALIX, CHMP4 and VPS4 during budding of HIV with abrogated Gag-ALIX interactions. ALIX interacts directly with HIV Gag through the YPXL late domain motif on Gag p6 [22,35,43]. In Gag (YP^−^) we abrogated this interaction by incorporating (_36_SR_37_) in place of (_36_YP_37_), as previously characterized [20]. Under these conditions, based on the canonical view, we were expecting to find reduced recruitment of ALIX into HIV Gag VLPs. Instead, we report observing multiple rounds of transient recruitment of ALIX, CHMP4 and VPS4 after completion of Gag assembly during virion budding. We further show that during each transient recruitment, the stoichiometry of all ESCRT components remained the same when compared to WT condition. We also show that the timely recruitment of ALIX to the budding VLP is dependent on the intact PTAP domain. Our results demonstrate that recruitment of ESCRTs is driven by a robust network of interactions resulting in an “on/off” switch behavior, and ALIX’s interactions with late domains of HIV Gag play a crucial role during final the final stages after assembly of the full ESCRT machinery.

## 2. Materials and Methods

### 2.1. Cell Culture and Transfection

HEK 293T, HeLa and U2OS cells were maintained in Dulbecco’s modified Eagle’s medium (DMEM; Invitrogen, Carlsbad, CA) supplemented with fetal calf serum (10%), sodium pyruvate (1 mM) and L-glutamine (2 mM). HeLa cell lines stably expressing eGFP-tagged ALIX were maintained in the same medium supplemented with G-418 (0.5 mg/mL) for selection. For TIRF experiments, cells were incubated in CO_2_-independent medium (LifeTechnologies, Grand Island, NY, USA).

Cells were seeded 18 h before transfection on sterile 4 chamber dishes at 60% confluence. Transfection was carried out using Lipofectamine2000 (Invitrogen, Carlsbad, CA, USA) and DNA plasmid at the ratio of 3:1 in HeLa and 2:1 for 293T cells and U2OS cells with total DNA of 1500 ng for imaging and 2000 ng for western blot. For stable cell line, the sample was supplemented in CO2-independent medium and moved to the microscope for imaging 4 h post transfection. In the case of transient co-transfection of DNA plasmids in normal HeLa, cells were used for imaging 6–7 h after transfection. The cells were kept at 37 °C during the imaging.

### 2.2. siRNA Transfections

HeLa cells were seeded at 40% confluence and were transfected 24 h later with siRNA targeting luciferase (CUGCCUGCGUGAGAUUCUCdTdT) or Alix (GAAGGAUGCUUUCGAUAAAUU) using Lipofectamine-2000. After 72 h, cells were re-transfected with siRNA. Again, after another 48 h cells were transfected with siRNA along with the desired plasmid. Cells were imaged 7 h later.

### 2.3. Microscopy

Live images were acquired using iMIC Digital Microscope made by TILL photonics controlled by TILL’s Live Acquisition imaging software as previously described [44] using Andor iXon camera. Two wavelengths of laser, 488 nm diode laser (Coherent, Saphire 488, Santa Clara, CA, USA) and 561 nm diode-pumped solid state (DPSS) laser (Cobolt Jive, 561 nm Jive High Power, San Jose, CA, USA), were used to excite eGFP and mCherry, respectively. 60× objective was used for the experiments. Laser beams passed through an AOTF (acousto-optical tunable filter) and focused into a fiber which delivers the light to TILL Yanus digital scan head and then Polytrope II optical mode switch. Polytrope hosts a quadrant photodiode used for TIRF penetration depth calibration, which was set to 150 nm for the experiments in this manuscript. Once the penetration depths for the experiments are set at the beginning of acquisition, a feedback loop keeps the focus of the objective on the sample by constantly monitoring the position of the back reflected beam with respect to the original beam.

### 2.4. Microscopy Data Analysis

Images from the microscope were stored as TIFF files and analyzed using Matlab software (Mathworks, Natick, MA, USA) as described previously [44]. The intensity of the fluorescent signal collected from each diffraction limited spot is proportional to the number of molecules within that position; however, the intensity is also proportional to the laser intensity, position of molecules with respect to glass during TIRF and substitution level of WT versus fluorescent molecules in each particular cell. To compare intensities of the ESCRT recruitments in between various cells and experimental conditions, average intensity (considering 25 VLPS from each cell) of the HIV unaffiliated ESCRT recruitments at the plasma membrane were used to normalize the fluorescent intensities in between cells. The intensity plots of VLPs are fitted using Boltzman growth equation. The timings of the recruitments were measured from the start of the Gag stationary phase to the intensity rise of the fluorescent ESCRTs. The later spikes are accounted in the histograms after adding them to the times of the first recruitments.

### 2.5. Cell Detachment Experiments

U2OS cells were transfected with Gag–eGFP or Gag–eGFP (YP^−^) and observed by TIRF imaging. At 12 h post-transfection, cells with VLPs were first imaged using TIRF and then cells were gently detached using TryplE (LifeTechnologies). Detachment was achieved by removing the medium and washing once with PBS; a thin layer of TryplE was added to cover cells to allow cell to detach. After a few minutes, the glass was again imaged with released VLPs left on the glass support.

### 2.6. Western Blot Analysis

Virion and cell lysates were separated on 4–15% polyacrylamide gels and transferred to Immobilon-FL membranes. Anti-p24 (183-H12-5C, NIH AIDS Reagent Program), anti–eGFP (Santa Cruz) and infrared dye coupled secondary antibodies (LI-COR) were used for immunoprobing. Scanning was performed with the Odyssey infrared imaging system (LI-COR) in accordance with the manufacturer’s instructions at 700 or 800 nm, accordingly.

### 2.7. Infectivity Assay

HEK 293T cells (60% confluent in 4 cm plates) were transfected using lipofectamine-2000 with NL4.3 vector alone or along with ΔCMV–eGFP–flex–CHMP4b plasmid. The supernatant was harvested 48 h later. Infectivity was measured by adding the supernatant to TZM-B1 cells (80% confluent); 48 h later cells were lysed using britelite plus Reporter Gene Assay (Perkin Elmer, Waltham, MA, USA) and luminosity was measured using a Cytation 5 microscope, experiments were carried out in triplicate.

### 2.8. Statistics

**Figure 1:**
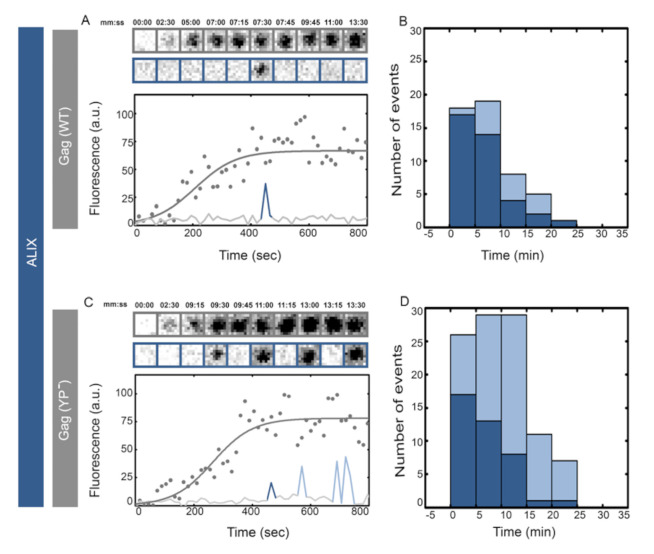
Single versus multiple transient recruitments of ALIX into HIV Gag VLPs versus HIV Gag (YP^−^) VLPs. HeLa cells stably expressing ALIX–h30–eGFP were transfected with 1500 ng of Gag–mCherry (**A**,**B**) or Gag (YP^−^)–mCherry (**C**,**D**) and imaged 4 h after transfection. Assemblies of individual representative VLPs are shown with intensity plots (as gray dots and fitted with gray line) and cropped TIRF images of the Gag (top, gray) and ALIX (bottom, Blue) for (**A**) Gag-mChery VLPs and (**C**) Gag (YP^−^)–mCherry VLPs. Histograms of the first time (dark blue) and later recruitments (light blue) of ALIX are shown for (**B**) Gag-mChery VLPs and (**C**) Gag (YP^−^)–mCherry VLPs. The majority of the first ALIX recruitment took place within 1–10 min after the VLP assembly completing during both YP^−^ and WT assembly.

**Figure 2:**
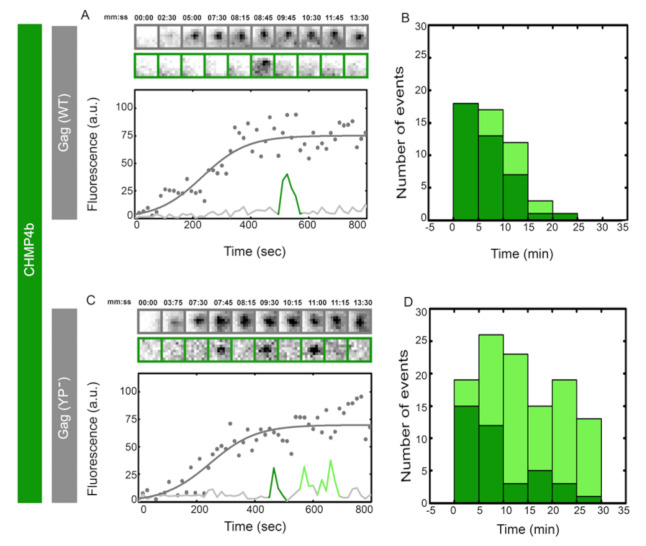
Single versus multiple transient recruitments of CHMP4 into HIV Gag VLPs versus HIV Gag (YP-) VLPs. HeLa cells were transfected with 900 ng of ΔCMV–eGFP–flex–CHMP4b and 600 ng (total 1500 ng plasmids) of Gag–mCherry (**A**,**B**) or Gag (YP-)–mCherry (**C**,**D**) and imaged 7 h after transfection. Assemblies of individual representative VLPs are shown with intensity plots (as gray dots and fitted with gray line) and cropped TIRF images of the Gag (top, gray) and CHMP4b (bottom, green) for (**A**) Gag–mCherry VLPs and (**C**) Gag (YP-)–mCherry VLPs. Histograms of the first time (dark green) and later recruitments (light Green) of CHMP4b are shown for (**B**) Gag–mCherry VLPs and (**C**) Gag (YP-)–mCherry VLPs. The majority of the first CHMP4b recruitment was within 1–10 min after the VLP assembly completed during both YP- and WT assembly.

**Figure 3:**
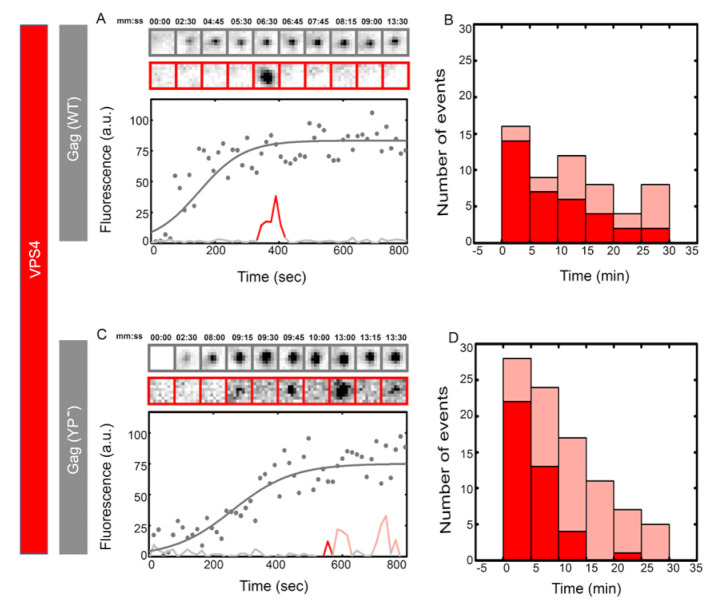
Single versus multiple transient recruitments of VPS4 into HIV Gag VLPs versus HIV Gag (YP-) VLPs.HeLa cells were transfected with 1200 ng of ΔCMV-VPS4-h37-mcherry and 300 ng (total 1500 ng of plasmids) of Gag–mCherry (**A**,**B**) or Gag (YP-)–mCherry (**C**,**D**) and imaged 7 h after transfection. Assemblies of individual representative VLPs are shown with intensity plots (as gray dots and fitted with gray line) and cropped TIRF images of the Gag (top, gray) and VPS4 (bottom, red) for (**A**) Gag–mCherry VLPs and (**C**) Gag (YP-)–mCherry VLPs. Histograms of the first time (dark red) and later recruitments (light red) of VPS4 are shown for (**B**) Gag-mChery VLPs and (**C**) Gag (YP-)–mCherry VLPs. The majority of the first VPS4 recruitment was within 1–10 min after the VLP assembly completed during both YP- and WT assembly.

**Figure 4:**
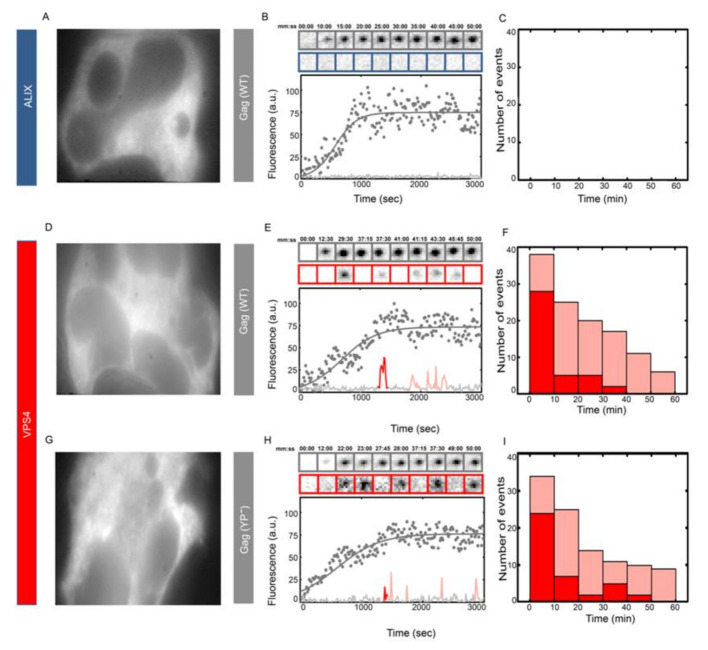
Multiple transient recruitments of VPS4 into HIV Gag WT and Gag (YP-) VLPs under ALIX depletion. HeLa cells were treated with two rounds of siRNA against ALIX. (**A**–**C**) HeLa ALIX–h37–eGFP cell line treated with siRNA against ALIX and then transfected with 1500 ng of Gag–mCherry WT. An individual multinucleated cell was chosen (**A**) and a representative HIV Gag–mCherry VLP assembly in this cell is shown in (**B**) with intensity plots (as gray dots and fitted with gray line) and cropped TIRF images of the Gag (top, gray) and ALIX (bottom, Blue). In 40 VLPs analyzed, there was no recruitment of ALIX, as shown in (**C**). HeLa cells were treated with siRNA against ALIX and then transfected with 1200 ng of ΔCMV–VPS4–h37–mCherry and 300 ng of HIV Gag–eGFP (**D**–**F**) and Gag (YP-)–eGFP (**G**–**I**). (**D**,**G**) Multinucleated cells chosen for experiments with (E&H) showing a representative HIV Gag–mCherry (**E**) or HIV Gag (YP-)–mCherry (**H**) VLP assembly with intensity plots and cropped TIRF images of the Gag (top, gray) and VPS4 (bottom, Red). (**F**,**I**) Histograms of the number and timing of the first (dark red) and later recruitment (light red) of VPS4 in (**F**) HIV Gag–mCherry VLPs and (**I**) HIV Gag (YP^−^)–mCherry VLPs.

All conditions tested contained 20 analyzed virus-like particles (supplementary Figures S1, S6 and S7) or 40 virus-like particles (main Figures 1–4) analyzed from 4–5 cells. The experiments were performed at least 3 times. There was no data selection applied to the sample, therefore all relevant data collected from the microscopy were analyzed and plotted in the figures.

### 2.9. Availability of Data

All data and reagents used in this study are available upon request; that includes the ΔCMV–eGFP–flex–CHMP4B plasmid characterized in this study and its sequence which is available upon request and also includes the Matlab code used for analysis of the imaging data.

### 2.10. Cell Lines

The HeLa, 293T and U2OS cells were obtained from ATCC. TZM-b1 cells were acquired from NIH AIDS Reagent Program.

## 3. Results

### 3.1. ALIX–h30–eGFP Molecules Are Recruited in Multiple Transient Events onto HIV Gag (YP^−^)–mCherry VLPs

We visualized the recruitment of ALIX during assembly of individual HIV Gag virus-like particles (VLPs) on the basal membranes of HeLa cells stably expressing ALIX–h30–eGFP. ALIX–h30–eGFP links ALIX with eGFP through a stiff 30 amino acid super helical linker and is functional with the same efficiency as WT in rescue of PTAP^−^ HIV virion release [44]. To generate fluorescent VLPs for fluorescent microscopy, cells were transfected with plasmids encoding HIV Gag–mCherry under a CMV promoter. Once VLP assembly commenced at the basal membrane, the membrane was imaged with a TIRF penetration depth of 150 nm using consecutive 488 and 561 nm illuminations every 15 s for 1.5 h (Section 2.3). Individual VLPs (showing sigmoidal curve in intensity rise) were identified and analyzed from their initiation until full assembly which corresponds to a stable fluorescence signal (fitted to the individual fluorescence intensities over time for a single VLP) from HIV Gag–mCherry, as shown in Figure 1. The releases of these VLPs are difficult to interpret as the released virions remain stuck to the glass. From 40 VLPs analyzed we detected 75% single transient recruitment of ALIX at the end of assembly and 25% showed an average of two transient recruitment events, as shown in Figure 1. The recruitments, as shown in cropped images, are depicted as rise in intensity level (spikes) in the figures.

ALIX interacts directly with HIV Gag through the YPXL late domain motif on Gag p6 [22,35,43]. As previously mentioned, in Gag (YP^−^) we abrogated this interaction by incorporating (_36_SR_37_) in place of (_36_YP_37_), as previously characterized [20]. We visualized the recruitment of ALIX during assembly of individual HIV Gag (YP^−^)–mCherry VLPs on the plasma membranes of cells stably expressing ALIX–h30–eGFP. Once VLP assembly commenced at the basal membrane, cells were imaged with identical settings to the imaging described above. The assembly times were not affected by the YP^−^ mutation as previously reported [44,45,46], and the average maximum intensities of HIV Gag (YP^−^)–mCherry VLPs were identical to wild type HIV Gag–mCherry VLPs (10,500 ± 4000 a.u. versus 12,000 ± 4000 a.u. respectively). After the completion of assembly, however, YP^−^ VLPs transiently recruited multiple rounds of ALIX, as shown in Figure 1. In 40 VLPs analyzed, 70% showed multiple rounds of recruitment “stuttering” with an average of four recruitment events per VLP. As shown in Figure 1, the arrival time of the first transient recruitment of ALIX in HIV Gag–mCherry VLPs was approximately 1–10 min post completion of the assembly, similarly to the arrival of the first transient recruitment of ALIX into HIV Gag (YP^−^)–mCherry VLPs. The tagging of Gag with mCherry had no effect on the observed ALIX recruitment phenotype since similar results were obtained in experiments where VLPs assembled with a mixture of HIV Gag (YP^−^)–mCherry along with HIV Gag (YP^−^), as shown in Figure S1.

The intensity of the maximum fluorescent signal is proportional to the number of ALIX–h30–eGFP molecules recruited to the sites of virion release. Based on our analysis with the time resolution used in our study, there was negligible difference between any transient ALIX recruitments into WT versus YP^−^ VLPs, as shown in Figure S2. To verify that YP^−^ VLPs had released from the host cells and did not remain tethered to the membrane, we detached the cells from the glass using incubation in TryplE (Section 2.5, [42]). Once the cells were removed the immobilized VLPs were visualized on the glass, as shown in Figure S3.

### 3.2. eGFP–Flex–CHMP4b Molecules Are Recruited in Multiple Transient Events onto HIV Gag (YP^−^)–mCherry VLPs

ALIX has a well-established biochemical interaction with CHMP4b through its Bro domain [7,20,21,22]. We visualized the recruitment of CHMP4b during the assembly of individual VLPs on the plasma membrane. To visualize this recruitment during HIV Gag VLP assembly, we created a plasmid which expresses human CHMP4b linked to eGFP by a flexible linker at its N-terminus under a ΔCMV promoter (ΔCMV–eGFP–flex–CHMP4b). A similar N-terminally tagged CHMP4b has been used before to visualize recruitment of CHMP4b onto the assembly of Gag VLPs by other laboratories [47,48]. The co-expression of this plasmid had no effect on the release of HIV Gag VLPs; we further characterized this plasmid in infectious HIV release and found a slight decrease in virion release with no effect on infectivity of the released virions [49] (Section 2.7).

We visualized the recruitment of CHMP4b during assembly of HIV Gag VLPs on the plasma membrane of HeLa cells co-transfected with ΔCMV–eGFP–flex–CHMP4b and HIV Gag–mCherry. Once VLP assembly commenced at the basal membrane, cells were imaged with identical settings to the imaging described above. Transient recruitment of CHMP4b was observed after completion of HIV Gag assembly, as shown in Figure 2, consistent with previous observations of CHMP4b recruitment into assembling VLPs [47]. From 40 VLPs analyzed, 80% showed a single CHMP4b recruitment and 20% showed an average of two CHMP4b transient recruitments.

We further visualized the recruitment of CHMP4b during the assembly of individual HIV Gag (YP^−^)–mCherry VLPs. As shown in Figure 2, CHMP4b was recruited transiently multiple times during assembly of HIV Gag (YP^−^) VLPs. From 40 VLPs analyzed, 75% showed multiple rounds of transient recruitment with an average of 4 recruitment events per VLP. The first transient recruitment of CHMP4b was 1–10 min after completion of HIV Gag assembly and was indistinguishable between Gag WT and YP^−^ VLPs. Similarly to the ALIX recruitment, the peak intensity of CHMP4b during transient recruitments had a negligible difference between WT and YP^−^ VLPs, as shown in Figure S4.

### 3.3. VPS4-h37–mCherry Molecules Are Recruited in Multiple Transient Events onto HIV Gag (YP^−^)–eGFP VLPs

At the late stages of ESCRT function, CHMP4b and VPS4 are recruited to catalyze the fission of the membrane. We further visualized the recruitment of VPS4 into WT versus HIV Gag YP^−^ VLPs, as shown in Figure 3. VPS4 proteins were linked to mCherry with a 37 amino acid long helical linker previously characterized [44]. HeLa cells were co-transfected with ΔCMV-VPS4-h37–mCherry along with HIV Gag–eGFP or HIV Gag (YP^−^)–eGFP, and assembly of HIV Gag VLPs and recruitment of VPS4 were recorded and analyzed as described above. In 40 WT VLPs analyzed, 70% showed a single VPS4 recruitment and 30% showed an average of two transient recruitments, as shown by another group too [41]. As shown in Figure 3, VPS4 is transiently recruited 1–10 min after completion of HIV Gag assembly with identical first recruitment timing between WT and YP^−^ VLPs. In 40 YP^−^ VLPs analyzed, 70% showed multiple rounds of VPS4 recruitment with an average of three recruitments per VLP. The peak intensity of VPS4 transient recruitments had a negligible difference between the WT versus YP^−^ condition, as shown in Figure S5. On calculating the residence time of all ESCRTs (ALIX, CHMP4b and VPS4), we found that their distribution does not vary much between WT and YP; the same can be concluded for the analysis of time between transient recruitments, as shown in Figure S6.

### 3.4. VPS4-h37–mCherry Molecules Are Recruited in Multiple Transient Events onto HIV NL4.3 (iGFP)(ΔENV)(YP^−^) VLPs

Our experiments up to this point were carried out using HeLa cells and the HIV Gag protein. To verify whether the observed stuttering of ESCRTs is more generally applicable and is not dependent on our particular experimental system we carried out the experiments in U2OS cells and also visualized the recruitment of VPS4 during assembly of NL4.3 (iGFP)(ΔENV) VLPs. We visualized the assembly of HIV Gag VLPs in U2OS cells by transfecting ΔCMV-VPS4-h37–mCherry plasmid along with HIV Gag–eGFP or HIV Gag (YP^−^)–eGFP into these cells. While the assemblies of both WT and YP^−^ VLPs in U2OS cells were slower, as shown in Figure S7, the recruitment of VPS4 showed a similar pattern in U2OS cells as in HeLa cells. We also measured the recruitment of VPS4 onto individual HIV virions assembling on the plasma membrane of HeLa cells by co-transfecting ΔCMV–VPS4–h37–mCherry and NL4.3 (iGFP)(ΔENV) or NL4.3 (iGFP)(ΔENV)(YP^−^). As shown in Figure S8, the observed stuttering of VPS4 during assembly of NL4.3 (iGFP)(ΔENV)(YP^−^) was similar to the stuttering observed during budding of HIV Gag (YP^−^)–eGFP VLPs.

### 3.5. Depletion of ALIX Using siRNA Results in Multiple Transient Recruitments of VPS4-h37–mCherry Onto HIV Gag (YP^−^)–eGFP VLPs

We further studied the effects of ALIX on the recruitment pattern of ESCRTs by depleting ALIX from cells using siRNA treatment. As shown in Figure S9, HeLa cells were depleted from ALIX after two rounds of the siRNA treatment that decreased the ALIX fluorescence by almost 75%. The depletion was further verified by the disappearance of the recruitment of ALIX–h30–eGFP onto HIV Gag and the accumulation of multinucleated cells due to ALIX depletion [32], as shown in Figure 4. After two rounds of siRNA treatment, multinucleated HeLa ALIX–h30–eGFP cells were transfected with HIV Gag–mCherry and the assembled HIV Gag–mCherry VLPs did not show any ALIX recruitment, as expected. However, under the same conditions, multinucleated HeLa cells transfected with ΔCMV-VPS4-h37–mCherry along with HIV Gag–eGFP or HIV Gag (YP^−^)–eGFP showed substantial stuttering of the recruitment of VPS4 for both HIV Gag constructs, as shown in Figure 4. The observed stuttering of VPS4 during budding of WT HIV Gag VLPs shows that the observed stuttering phenotype is present due to critical function of ALIX which is missing in both cells depleted of ALIX and in VLPs formed by the YP- mutated HIV Gag.

### 3.6. ALIX–h30–eGFP Molecule Are Recruited Transiently With a Substantial Delay onto Gag (PTAP^−^)–mCherry and Gag (PTAP^−^ + YP^−^)–mCherry VLPs

**Figure 5:**
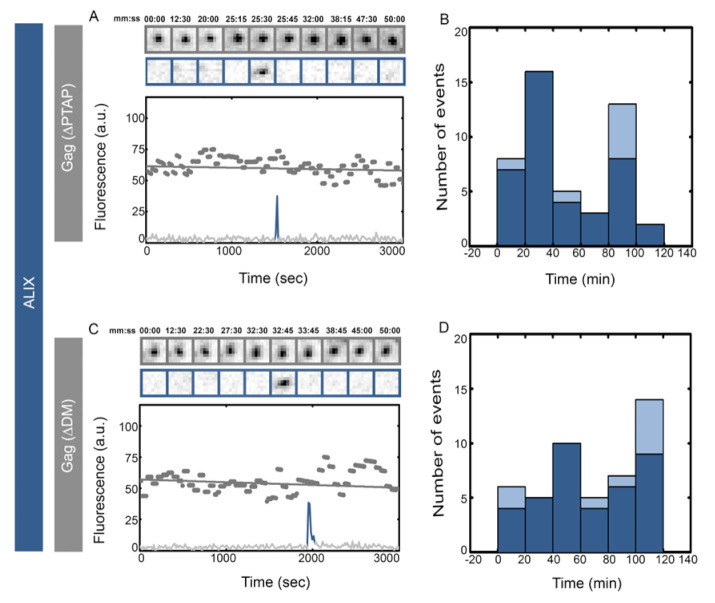
Delayed transient recruitment of ALIX into HIV Gag (YP^−^ + PTAP^−^) VLPs or HIV Gag (PTAP^−^) VLPs. HeLa cells stably expressing ALIX–h30–eGFP were transfected with 1500 ng of Gag (PTAP^−^)–mCherry (**A**,**B**) or Gag (PTAP^−^ + YP^−^)–mCherry (**C**,**D**) and imaged 5 h after transfection. Intensity plots (as gray dots and fitted with gray line) of individual fully assembled representative VLPs are shown and cropped TIRF images of the Gag (top, gray) and AIX (bottom, blue) for (**A**) Gag(PTAP^−^)–mCherry VLPs and (**C**) Gag (PTAP^−^ + YP^−^)–mCherry VLPs. Histograms of the first time (dark blue) and later recruitments (light blue) of ALIX are shown for (**B**) Gag (PTAP^−^)–mCherry VLPs and (**C**) Gag (PTAP^−^ + YP^−^)–mCherry VLPs. Alix was recruited within 2 h after the start of the actual experiment.

There are two late motifs identified in the HIV Gag-p6 domain. The PTAP motif has been shown to directly interact with TSG101 and is critical for release of infectious HIV virions from infected cells [13,14,15]. We further visualized the recruitment of ALIX into HIV Gag VLPs with both PTAP^−^ which incorporates a (_7_LIRL_10_) instead of (_7_PTAP_10_) [11] and PTAP^−^ + YP^−^ which has both PTAP and YP sequences altered (_7_LIRL_10_ plus _36_SR_37_). HeLa cells stably expressing ALIX–h30–eGFP were transfected with HIV Gag (PTAP^−^)–mCherry or HIV Gag (PTAP^−^ + YP^−^)–mCherry, and assembly kinetics and recruitment of ALIX were visualized with TIRF imaging, as described above. During the initial experiments conducted similarly to previously described experiments, no recruitment events were observed on the HIV Gag VLP assembly sites. To probe a later time frame recruitment, we therefore initiated the TIRF imaging one hour after the initial formation of VLPs. In these sets of experiments, the VLPs were already fully assembled at the initiation of plasma membrane imaging and therefore exact timing of recruitment events to the completion of assembly time could not be deduced. These experiments showed single transient recruitment of ALIX–h30–eGFP at a time frame between 1 h and 2 h post assembly of VLPs, as shown in Figure 5. From 40 VLPs analyzed, 80% had a single recruitment event and 20% showed an average of two recruitment events. The multiple recruitments observed in the YP^−^ VLPs were not observed in the PTAP^−^ VLPs. The transient recruitment of ALIX was identical between PTAP^−^ and PTAP^−^ + YP^−^ mutation, as shown in Figure 5. The intensity of the ALIX–h30–eGFP in transient recruitments had a negligible difference between WT, PTAP^−^ and PTAP^−^ + YP^−^ VLPs, as shown in Figure S10.

## 4. Discussion

The recruitment of ALIX, CHMP4b and VPS4 with almost the same number of molecules under various late domain mutations argues that recruitment of ESCRTs is driven by a cooperative network which can be triggered through multiple entry points with identical net resulting recruitment (Figures S2, S4 and S5). More recently, binding of ubiquitin to Gag through ubiquitin ligases and membrane curvature were shown to play roles in recruitment of ESCRTs [50,51,52,53,54,55]; however, how these events are choreographed on the plasma membrane remains to be explored.

HIV-1 mainly infects CD4^+^ helper T cells and macrophages in vivo [56,57]. Here, we have mostly used HIV Gag constructs and performed the experiments in HeLa cells because of their ideal membrane configuration for imaging purposes and the mostly static phenotype of Gag VLPs building on their membrane. This system has allowed easy tracking and observation of multiple rounds of ESCRT recruitment in the same VLP. Observing multiple rounds of assembly during HIV budding in T-cells remains technically out of reach, due to the limited membrane and cellular movements of T-cells [58,59].

Multiple VPS4 recruitments are rarely observed after completion of Gag WT assembly, and even when they are observed, no more than two recruitments are observed on the same VLP [41,47,60] and Figure 1, Figure 2, Figure 3 and Figure 4. The stuttering recruitment of the full ESCRT machinery which is characterized by more than three recruitments of the ESCRT machinery in the same YP^−^ VLPs is therefore significant and novel and suggests that ALIX plays a major role during end stages of ESCRT function. We hypothesize that the catastrophic disassembly of all of the ESCRT machinery during stuttering on the YP- VLPs indicates that a failure of ALIX to connect properly with Gag results in a catastrophic disassembly of all ESCRT components. This disassembly is then followed by re-assembly of the full ESCRT machinery, resulting in stuttering recruitment of ESCRTs. The molecular mechanism and the ultra-structural localizations of all the ESCRT machinery during membrane fission are unclear and would require significant new investigations.

We also show that PTAP mutation delays but does not stop the recruitment of ALIX. Therefore, we argue that the prevalent linear biochemical interaction map between ESCRTs may unnaturally simplify the in vivo function of these interactions. There are up to five different ALIX interactions functioning at late stages of VLP assembly: (i) a direct ALIX–Gag interaction through the YPXL late domain motif on Gag p6 [22,35,43], (ii) a direct ALIX–Gag interaction through a binding site on Gag NC [61,62,63], (iii) ALIX interactions with ubiquitin [64,65], (iv) ALIX–TSG101 interactions [20,21,66] and (v) interactions with ALIX itself, including relief of ALIX autoinhibition [67,68],opening of the V domain [67] and possibly ALIX dimerization [69]. Our results suggest that the exact choreography of these interactions and what role they play during the function of the full ESCRT machinery is not simply recruitment and remains to be visualized in vivo.

We have previously shown that mutations within the late domain of HIV result in a delayed release of the virus, which in turn results in budding of non-infectious virions due to premature protease activation [42]. These kinetic biochemical assays showed an approximate delay of 20 min for HIV Gag (YP^−^) in U2OS cells and 70 min for fully infectious virions with YP^−^ mutation [42]. This delay is consistent with the time between the first recruitment of ESCRTs and the last recruitment of ESCRTs on individual VLPs visualized in this study. Our measurements show an average time between these recruitments as 10 ± 8 min for HIV Gag (YP^−^) in HeLa cells, 18 ± 13 min for HIV Gag (YP^−^) in U2OS cells and 37 ± 45 min for NL4.3 (iGFP)(ΔENV)(YP^−^) in HeLa cells. Recent studies have shown recruitment of ESCRTs is followed by release of the virion within a 20 s time window from the last ESCRT recruitment [41]. Therefore, we suggest that in our experiments, virion release happened after the last recruitment of ESCRTs after the stuttering events on individual virions. During imaging of HIV virus-like particle (VLP) budding from mammalian cells in culture, membrane fission was detected to occur up to a minute after all the ESCRTs had been released back into the cytosol [41]. In our study we did not directly measure viral release; we only report a constant time delay in release of Gag VLPs with the delay associated with stuttering. We therefore cannot report on the exact moment of virion release with respect to the last ESCRT recruitment in our study. Experiments to visualize both the fluorescence recruitment and virion release are, however, the focus of future work.

The ALIX homologue Bro1 in yeast is proposed to be recruited through interactions with Snf7, a yeast homologue of CHMP4 [6,7]. ALIX has a Bro1 domain analogous to the yeast Bro1, along with a V domain and a PRR which does not exist in the yeast homologue Bro1 [7,22]. The binding of late domain YPXL has been mapped to the ALIX V domain [22] and Cepp55 binds the PRR [30,32]. Such apparent diversity had suggested that the recruitment and possibly function of ALIX is evolutionarily separate from the yeast homologue Bro1. In contrast to this view, our observations showing that the late domain does not play a role in recruitment of ALIX is more in agreement with the findings in yeast where Bro1 was shown to regulate the function of ESCRT-III protein Snf7 during membrane scission [70]. While ALIX has been shown to be important in function of ESCRTs in all membrane scission reactions, a unified understanding of its function has been lacking. Based on all above data and available literature we suggest that ALIX plays a critical role during the final stages of membrane fission along with ESCRT-III and VPS4 proteins.

Live imaging of HIV and MVB budding has been previously used for visualizing recruitment of ESCRTs during membrane scission events [40,41,44,47,60,71,72,73]. Our study shows how disturbing previously characterized biochemical interactions can result in surprising recruitment profiles of ESCRTs observed in live cells and therefore underscores the usefulness of the imaging methods for further characterizing these interactions in vivo.

## Supplementary Materials

The following are available online at https://www.mdpi.com/1999-4915/12/9/1032/s1. Figure S1. Similar multiple transient recruitments of ALIX into HIV Gag VLPs with mixture of tagged and untagged Gag. Figure S2. Distribution of the maximum fluorescence intensity of ALIX transient recruitment events. Figure S3. Similar release of Gag WT and (YP^−^) VLPs. Figure S4. Distribution of the maximum fluorescence intensity of CHMP4 transient recruitment events. Figure S5. Distribution of the maximum fluorescence intensity of VPS4 transient recruitment events. Figure S6. Distribution of the retention time of ESCRT and the time between ESCRT spikes into Gag (WT) or Gag (YP^−^) VLPs. Figure S7. Recruitment of VPS4 into HIV Gag and HIV Gag (YP^−^) VLPs in U2OS cells. Figure S8. Transient recruitment of VPS4 into NL4.3 (iGFP)(ΔENV) versus NL4.3 (iGFP)(ΔENV) (YP^−^) VLPs. Figure S9. ALIX depletion using siRNA. Figure S10. Distribution of the maximum fluorescence intensity of ALIX transient recruitment events into (PTAP^−^) and (PTAP^−^ + YP^−^) VLPs.
