# Supplementary material for: Abrogating ALIX Interactions Results in Stuttering of the ESCRT Machinery
Source: Viruses. 2020 Sep 16;12(9):1032. doi: 10.3390/v12091032 (PMC7551432; doi:10.3390/v12091032)
Supplement: Supplementary file 1 [file viruses-12-01032-s001.pdf]

viruses

Supplementary Materials

Supplementary Figures

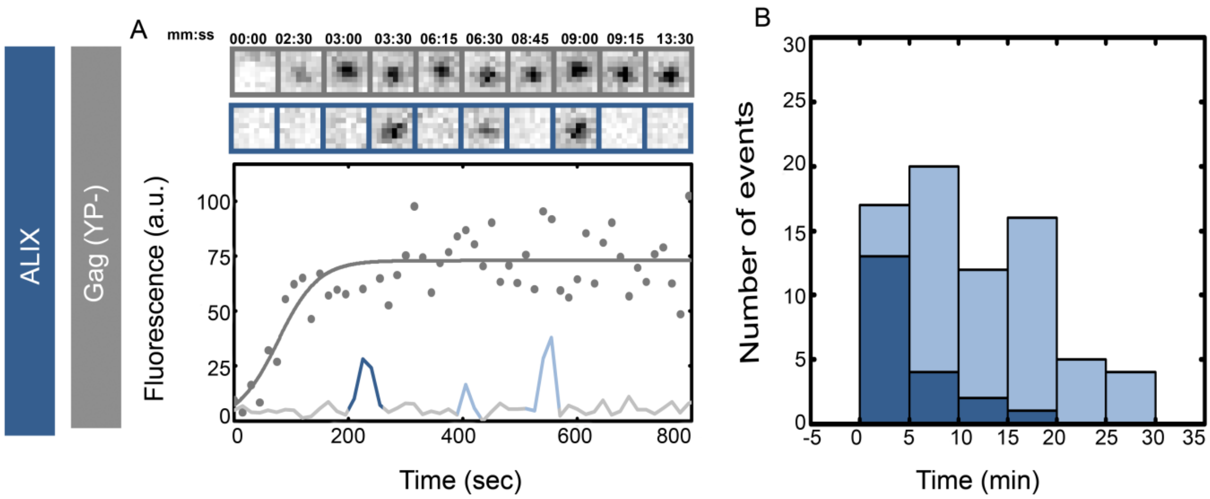

Figure S1. Similar multiple transient recruitments of ALIX into HIV Gag VLPs with mixture of tagged and untagged Gag**.** HeLa cells stably expressing ALIX-h30-eGFP were transfected with 300 ng of Gag (YP^{-}) and 1200 ng of Gag (YP^{-})-mCherry and imaged 5 hours after transfection. (**A**) Shows assembly of individual representative VLPs with intensity plots (as Gray dots and fitted with Gray line) and cropped TIRF images of the Gag (Top, Gray) and ALIX (bottom, Blue). Histograms of the first time (dark blue) and later recruitments (light blue) of ALIX are shown in (**B**). Majority of the first ALIX recruitment is within 1-10 minutes after the VLP assembly completes during YP^{-} assembly.

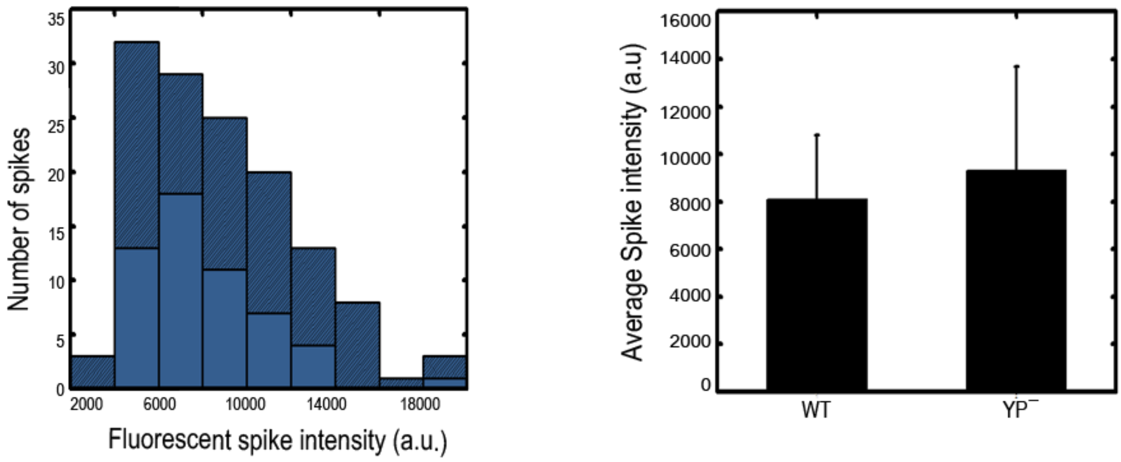

Figure S2. Distribution of the maximum fluorescence intensity of ALIX transient recruitment events**.** The maximum fluorescence intensity detected during recruitment is proportional to the number of molecules recruited to the site of assembly. Figures show a comparison between intensities during recruitment into HIV Gag-mCherry VLPs (light blue) versus HIV Gag (YP^{-})-mCherry VLPs (dark blue).

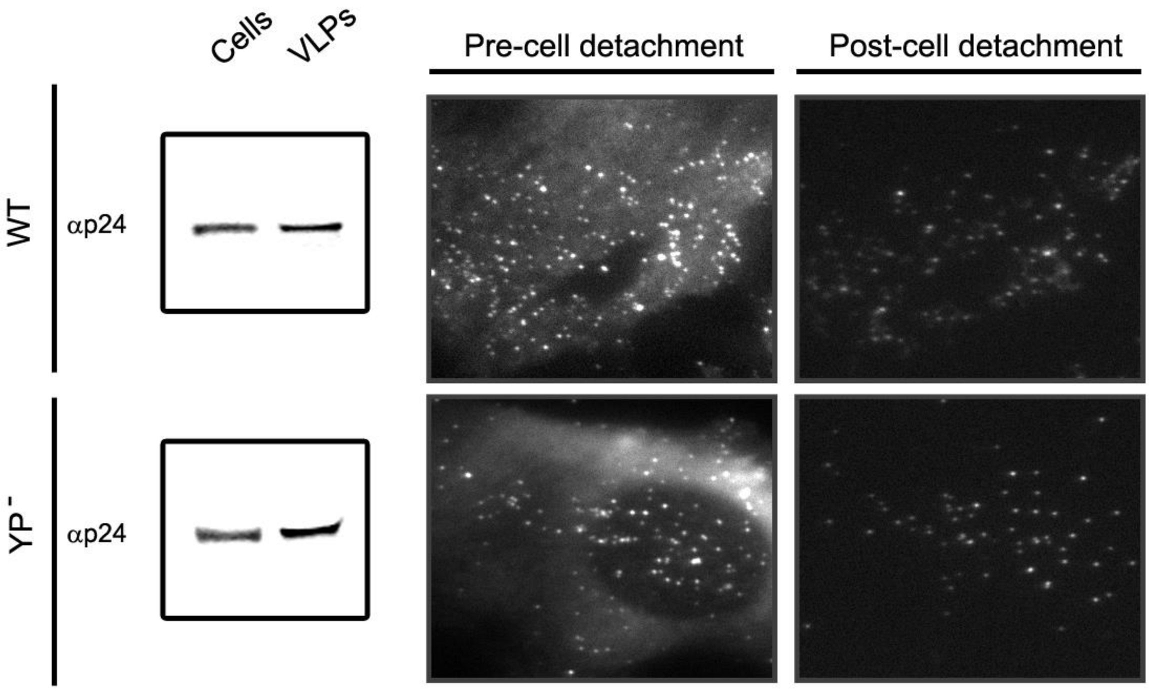

Figure S3. Similar release of Gag WT and (YP^{-}) VLPs. Western blot conducted 24 hours after transfecting U2OS cells with Gag WT or (YP^{-}) and immunoprobed with p24 (left panel) which shows no difference in release of VLPs from cells transfected with HIV Gag or HIV Gag (YP^{-}). Single U2OS cells were imaged 12 hours post-transfection of HIV Gag-eGFP WT or HIV Gag (YP^{-})-eGFP using TIRF microscopy before and after cell detachment to visualize released VLPs (right panels).

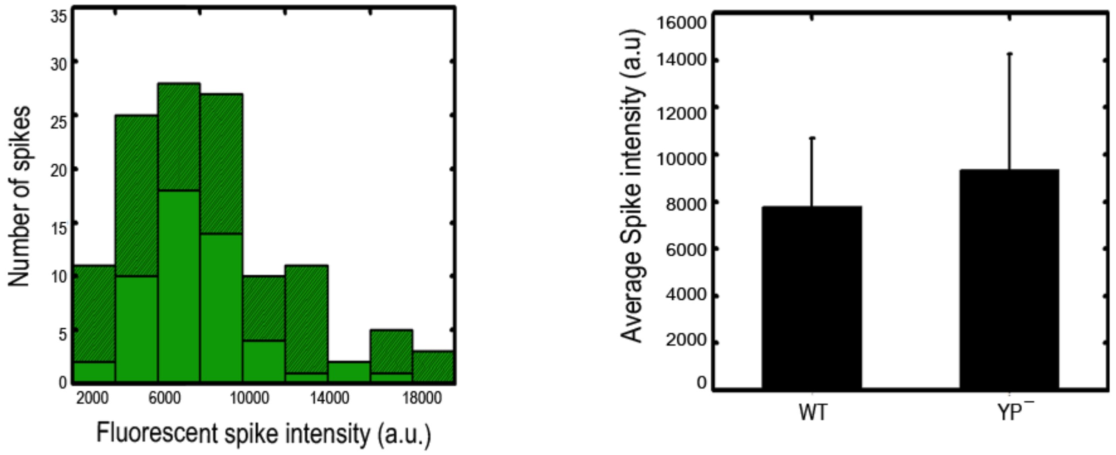

Figure S4. Distribution of the maximum fluorescence intensity of CHMP4 transient recruitment events**.** The maximum fluorescence intensity detected during recruitment is proportional to the number of molecules recruited to the site of assembly. Figures show a comparison between intensities during recruitment into HIV Gag-mCherry VLPs (light green) versus HIV Gag (YP$^-$)-mCherry VLPs (dark green).

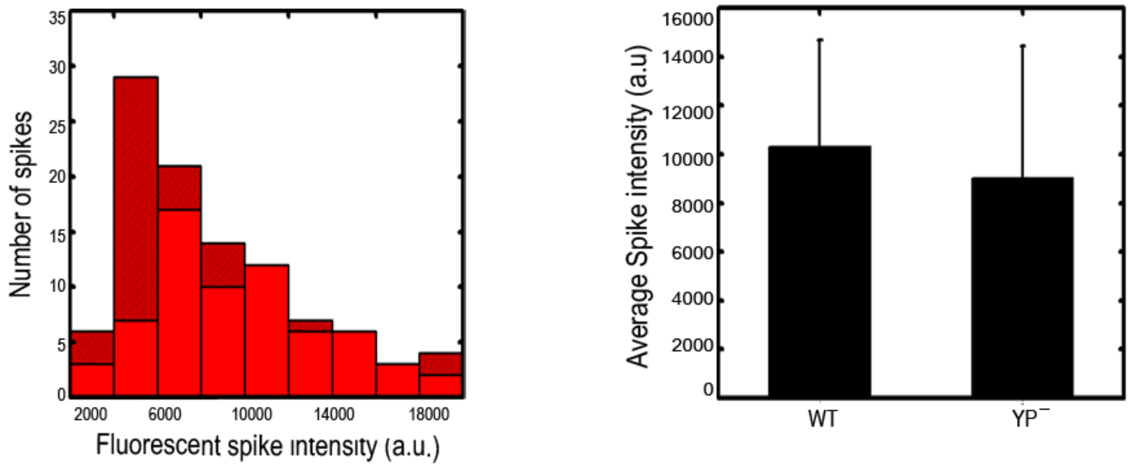

Figure S5. Distribution of the maximum fluorescence intensity of VPS4 transient recruitment events**.** The maximum fluorescence intensity detected during recruitment is proportional to the number of molecules recruited to the site of assembly. Figures show a comparison between intensities during recruitment into HIV Gag-mCherry VLPs (light red) versus HIV Gag (YP$^-$)-mCherry VLPs (dark red).

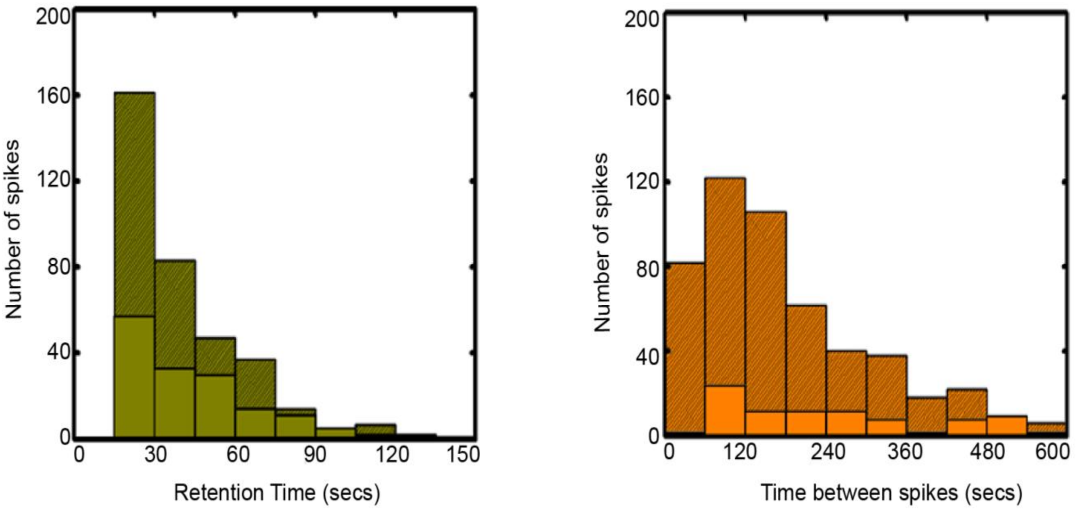

Figure S6. Distribution of the retention time of ESCRT and the time between ESCRT spikes into Gag (WT) or Gag (YP^{-}) VLPs. Figure (left) show a comparison between time of residence at the membrane recruitment site into Gag (WT) VLPs (light green) versus Gag (YP^{-}) VLPs (dark green). Figure (right) show a comparison between time of spike interval at the membrane recruitment site into Gag (WT) VLPs (light orange) versus Gag (YP^{-}) VLPs (dark orange).

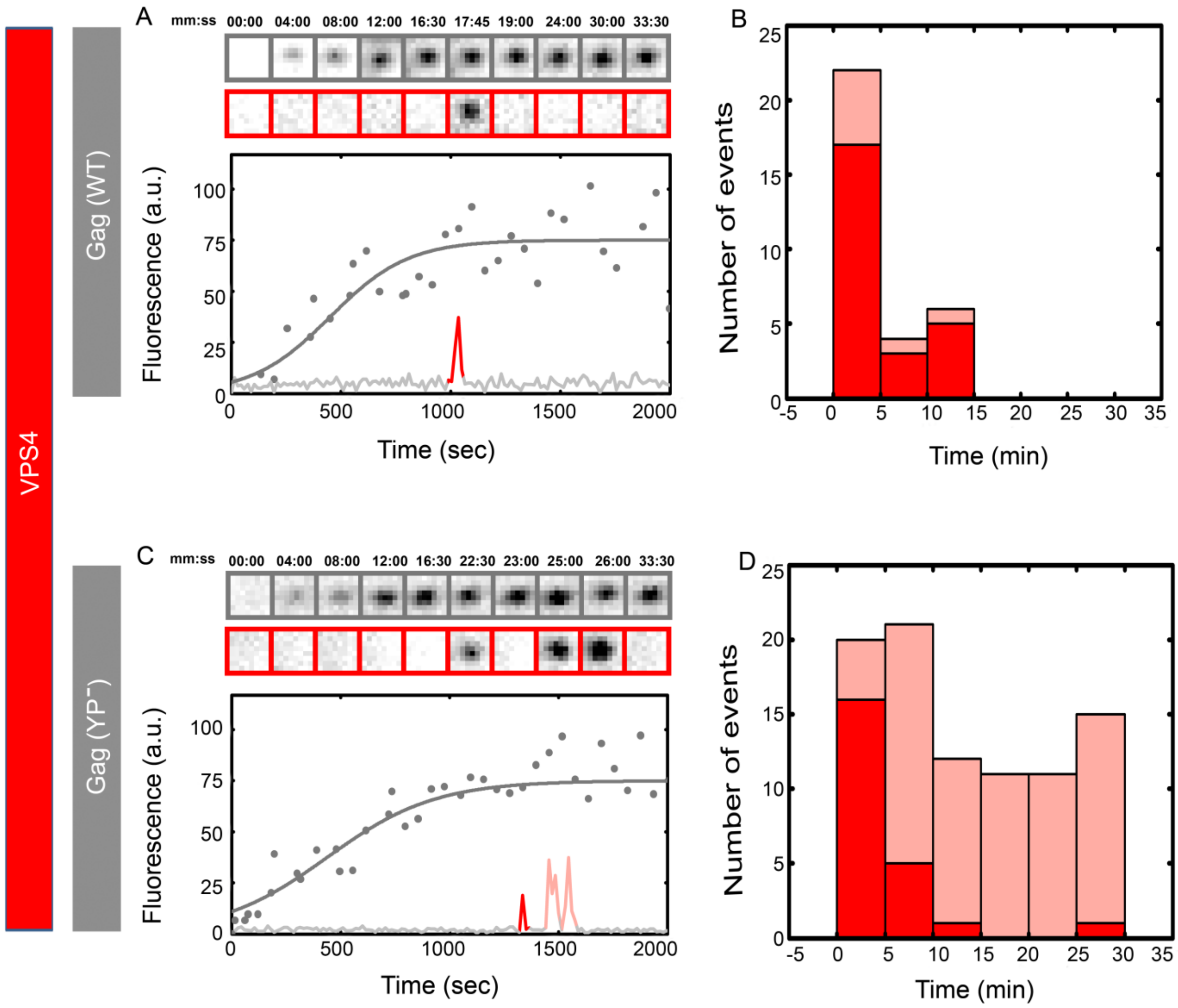

Figure S7. Recruitment of VPS4 into HIV Gag and HIV Gag (YP-) VLPs in U2OS cells. U2OS cells were transfected with 1200 ng of CMV-VPS4-h37-mcherry and 300 ng of Gag-mCherry (**A** and **B**) and Gag (YP-)-mCherry (**C** and **D**) and imaged 7 hours after transfection. Assembly of individual representative VLPs are shown with intensity plots (as Gray dots and fitted with Gray line) and cropped TIRF images of the Gag (Top, Gray) and VPS4 (bottom, Red) for (**A**) Gag-mChery VLPs and (**C**) Gag (YP-)-mCherry VLPs. Histograms of the first time (dark Red) and later recruitments (light Red) of VPS4 are shown for (**B**) Gag-mChery VLPs and **(C)** Gag (YP-)-mCherry VLPs. Majority of the first VPS4 recruitment is within 1–10 minutes after the VLP assembly completes during both YP- and WT assembly.

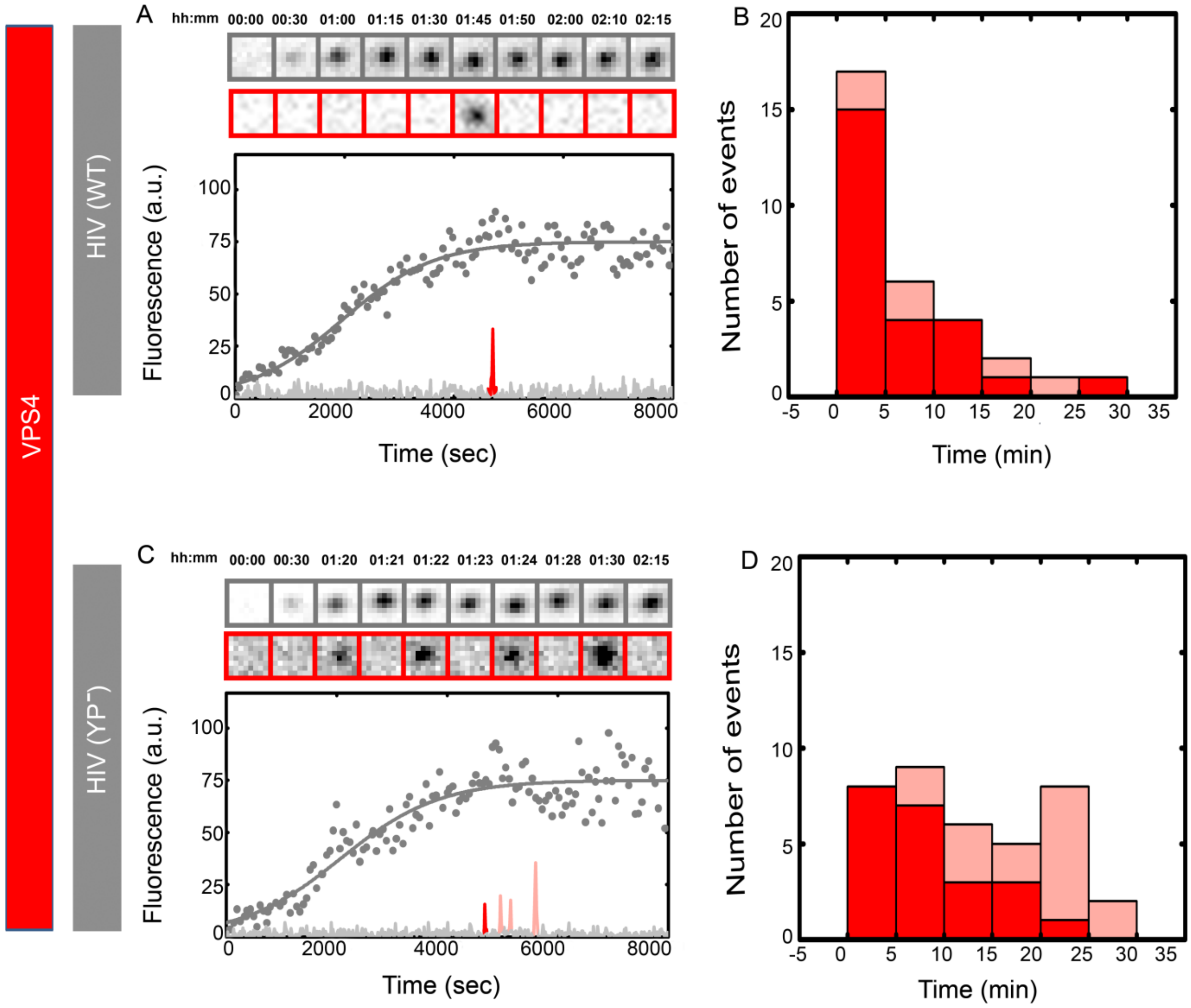

Figure S8. Transient recruitment of VPS4 into NL4.3 (iGFP)(ΔENV) versus NL4.3 (iGFP)(ΔENV) (YP^{-}) VLPs. Hela cells were transfected with 300 ng of ΔCMV-VPS4-h37-mcherry and 1200 ng of NL4.3 (iGFP)(ΔENV) (**A** and **B**) and NL4.3 (iGFP)(YP^{-})(ΔENV) (**C** and **D**) and imaged 12 hours after transfection. Assembly of individual representative VLPs are shown with intensity plots (as Gray dots and fitted with Gray line) and cropped TIRF images of the Gag (Top, Gray) and VPS4 (bottom, Red) for (**A**) NL4.3 (iGFP)(ΔENV) VLPs and (**C**) NL4.3 (iGFP)(YP^{-})(ΔENV) VLPs. Histograms of the first time (dark Red) and later recruitments (light Red) of VPS4 are shown for (**B**) NL4.3 (iGFP)(ΔENV) VLPs and (**C**) NL4.3 (iGFP)(YP^{-})(ΔENV). Majority of the first VPS4 recruitment is within 1-20 minutes after the VLP assembly completes during both YP^{-} and WT assembly.

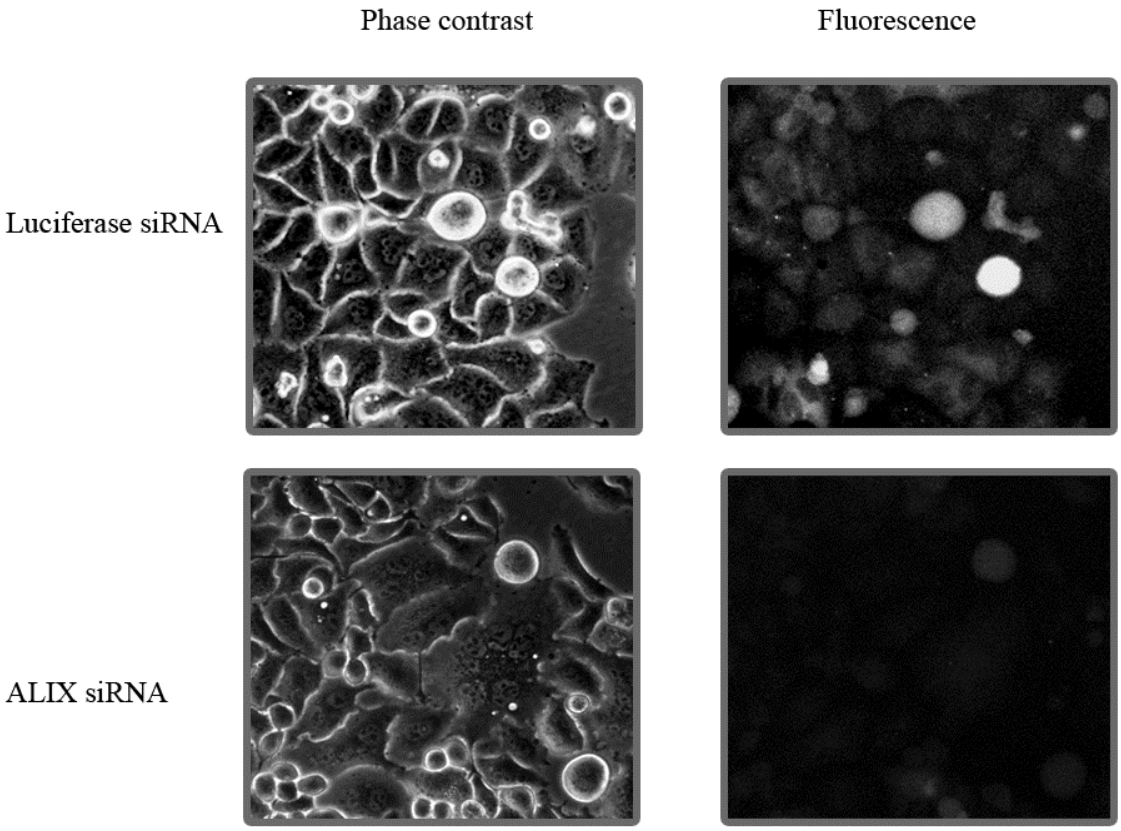

Figure S9. ALIX depletion using siRNA. Hela ALIX-h30-eGFP cells were treated with siRNA against ALIX or siRNA against luciferase (control) and imaged after 6 days. Fluorescence decrease and multinucleated cells are visualized in siRNA treatment against ALIX.

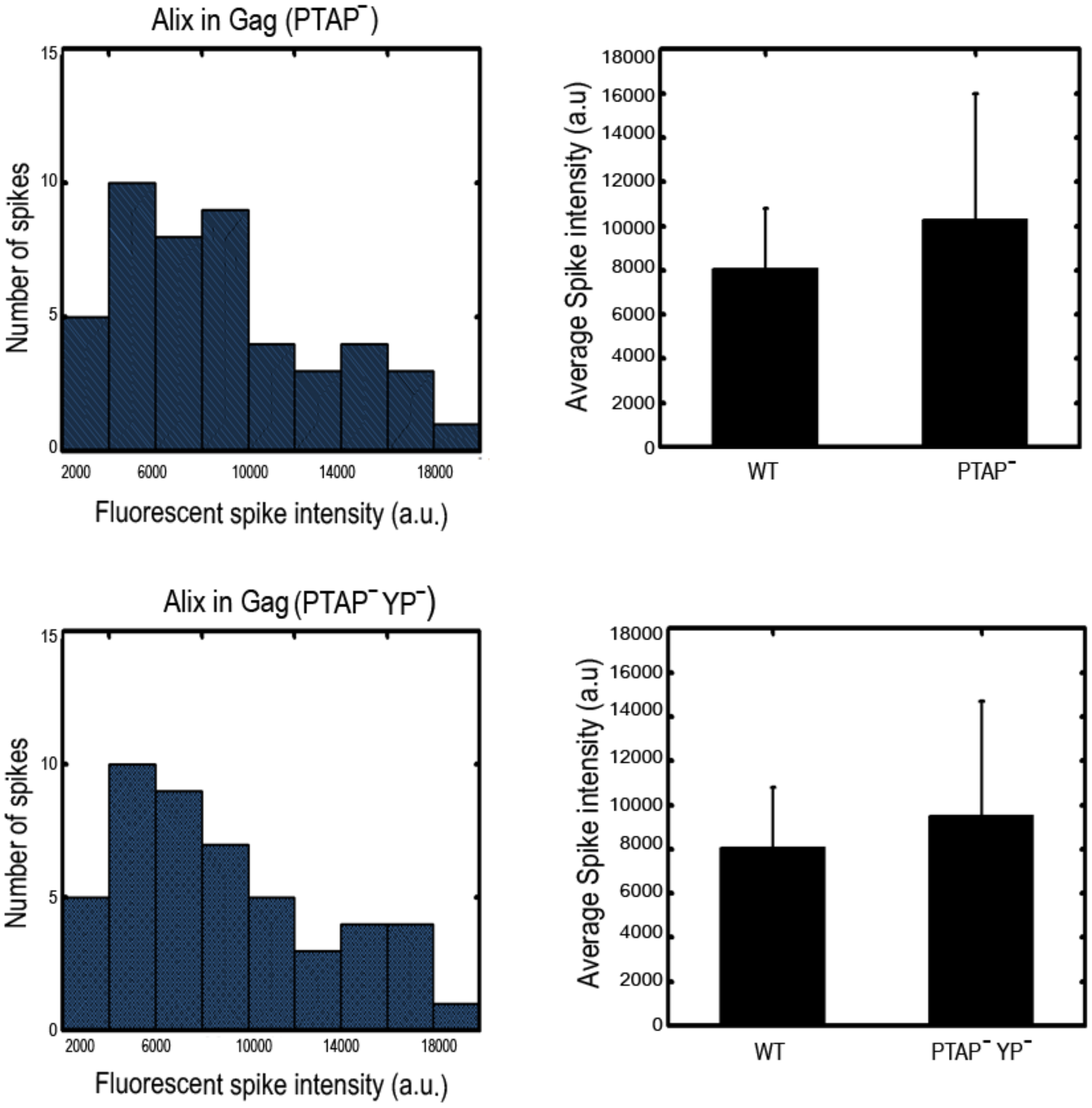

Figure S10. Distribution of the maximum fluorescence intensity of ALIX transient recruitment events into (PTAP$^-$) and (PTAP$^-$ + YP$^-$) VLPs**.** The maximum fluorescence intensity detected during recruitment is proportional to the number of molecules recruited to the site of assembly.
